# Preference for novel faces in male infant monkeys predicts cerebrospinal fluid oxytocin concentrations later in life

**DOI:** 10.1038/s41598-017-13109-5

**Published:** 2017-10-11

**Authors:** Jesus E. Madrid, Ozge Oztan, Valentina Sclafani, Laura A. Del Rosso, Laura A. Calonder, Katie Chun, John P. Capitanio, Joseph P. Garner, Karen J. Parker

**Affiliations:** 1 Neurosciences Program, Stanford University, Stanford, CA 94305 USA; 2 Department of Psychiatry and Behavioural Sciences, Stanford University, Stanford, CA 94305 USA; 3 California National Primate Research Centre, University of California Davis, Davis, CA 95616 USA; 4 Winnicott Research Unit, University of Reading, RG6 6AL Reading, UK; 5 Department of Psychology, University of California Davis, Davis, CA 95616 USA; 6 Department of Comparative Medicine, Stanford University, Stanford, CA 94305 USA

## Abstract

The ability to recognize individuals is a critical skill acquired early in life for group living species. In primates, individual recognition occurs predominantly through face discrimination. Despite the essential adaptive value of this ability, robust individual differences in conspecific face recognition exist, yet its associated biology remains unknown. Although pharmacological administration of oxytocin has implicated this neuropeptide in face perception and social memory, no prior research has tested the relationship between individual differences in face recognition and endogenous oxytocin concentrations. Here we show in a male rhesus monkey cohort (N = 60) that infant performance in a task used to determine face recognition ability (specifically, the ability of animals to show a preference for a novel face) robustly predicts cerebrospinal fluid, but not blood, oxytocin concentrations up to five years after behavioural assessment. These results argue that central oxytocin biology may be related to individual face perceptual abilities necessary for group living, and that these differences are stable traits.

## Introduction

A fundamental challenge confronted by individuals living in social groups is the ability to recognize others. In visually-dependent primate species characterized by extensive social interactions, the ability to recognize faces is important for individual identification and is necessary for the development of preferential relationships that enhance survival^1–7^. Despite the crucial role of face recognition ability in primate societies, there is nevertheless within-species variation in the ability to recognize faces, which is evident from an early age^8,9^, and is associated with long-lasting differences in social development^10^.

Recent research has begun to elucidate the neurobiological mechanisms underlying the ability to recognize faces^11,12^. One neurotransmitter thought to mediate face processing in primates is oxytocin (OT), which has an evolutionarily-conserved involvement in the regulation of social recognition, social reward, social bond formation, and parental care across taxa^13–16^. More specifically, OT administration in human and non-human primates increases attention to the eye region^17^ and to faces^18–20^, by changing an individual’s gaze fixation and saccade patterns^21,22^. These effects, in turn, may underlie the consistently observed improvements in face memory following OT administration^23–27^.

The majority of research investigating the role of OT in primate face processing has relied on intranasal administration of OT. Exogenously administered OT, however, induces OT concentrations that exceed normal physiological ranges^28–30^, which may or may not mimic the messaging mechanisms of endogenously released OT^31–35^. Our understanding of the behavioural effects of OT at physiological concentrations is thus severely lacking^15^. Consequently, no studies to date have tested whether naturally occurring variation in face processing is related to endogenous OT concentrations.

In order to understand whether naturally occurring variation in primate face processing is related to endogenous OT concentrations, we must also determine the most meaningful biological medium in which to measure it. OT is produced in hypothalamic neurons and delivered to brain nuclei and into the ventricular system via central pathways; it is also released into peripheral blood circulation via the posterior pituitary^36^. Once released into peripheral circulation, OT does not cross the blood-brain barrier in significant amounts^37^. Most studies of OT biology in human and non-human primates have relied on peripheral OT measurement (e.g., from blood, saliva, or urine) because these readily accessible media require less invasive sampling methods compared to those used for central OT measurement (e.g., from extracellular neuronal dialysate, or cerebrospinal fluid [CSF])^38^. Peripheral OT concentrations are most, or arguably only, informative, however, if they predict behaviourally relevant central OT concentrations. Yet, the relationship between blood and CSF OT concentrations in non-human primate species remains poorly tested to date.

The present study addressed several fundamental gaps in knowledge by testing the relationship between individual variation in a face recognition task and endogenous OT biology, as well the relationship between peripheral and central OT measurements. Specifically, we assessed preference for novel faces in infant rhesus macaques (*Macaca mulatta*), using a standardized face recognition test when monkeys were 3–4 months of age^39^. We next collected CSF and blood samples when subjects were between 1–5 years of age; samples were subsequently quantified for OT concentrations. Here we show that performance on the face recognition test significantly and positively predicted CSF OT concentrations up to five years after behavioural assessment. Behavioural performance did not predict blood OT concentrations, and blood and CSF OT measurements were unrelated within individuals. To our knowledge these are the first data linking individual differences in face processing to endogenous OT biology in primates, a relationship that spans juvenility into adulthood. Furthermore, these data suggest the importance of measuring CSF, rather than blood, OT concentrations when addressing social perception traits in primates.

## Results

### Variation in preference for novel faces

**Figure 1:**
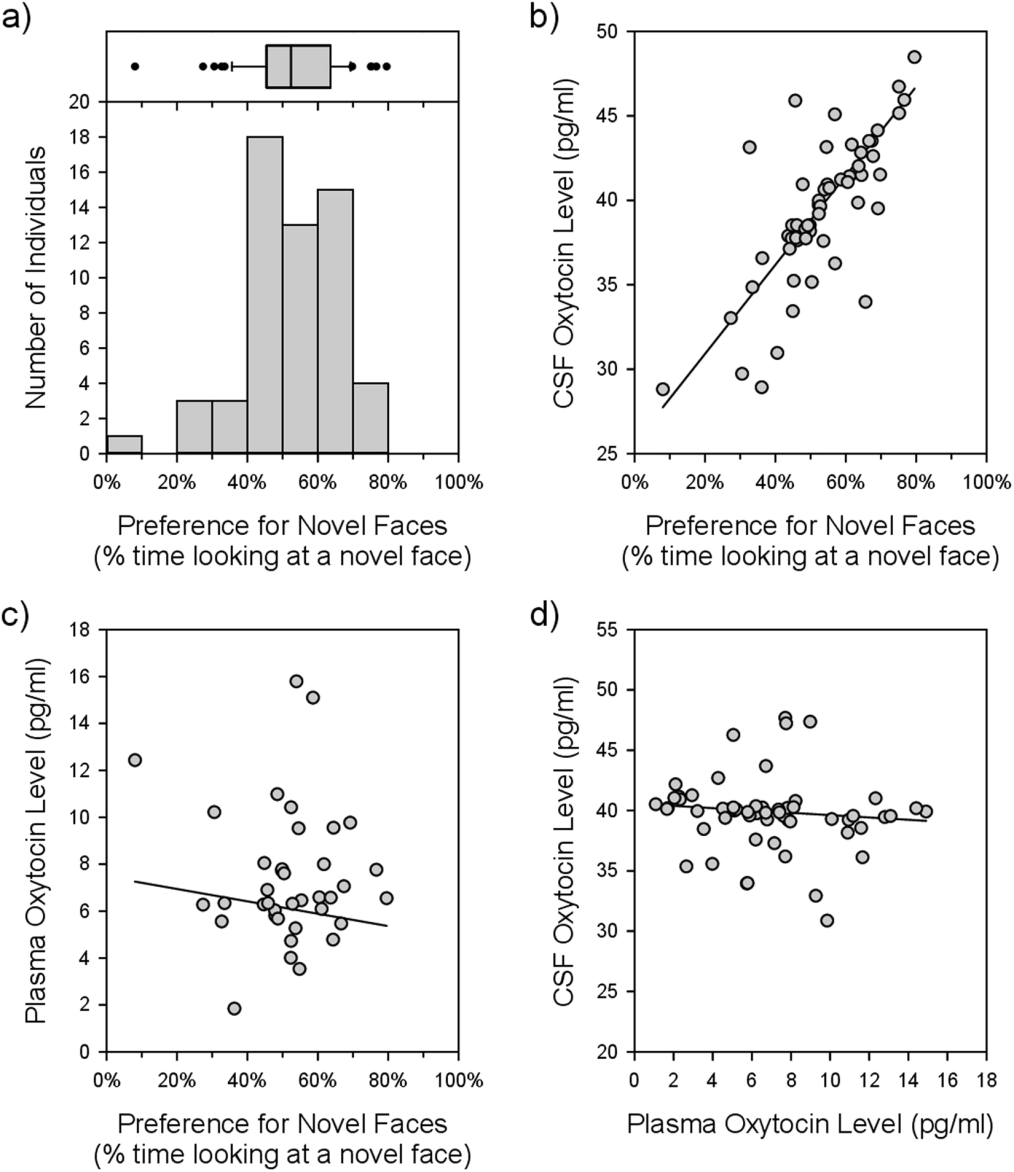
Infant preference for novel faces and later measures of oxytocin (OT) biology. (**a**) Individual variation in visual preference for novel faces. The box-and-whisker plot shows the interquartile range and median (box), and the 10^th^ and 90^th^ percentiles (whiskers), and remaining outliers. (**b**) Early preference for novel faces predicts later cerebrospinal fluid (CSF) OT concentrations (P = 0.0048). CSF OT concentrations are corrected to reflect the Weighted Least Squares General Linear Model (WLS-GLM) analysis, and are thus plotted as the expected value for each data point, plus the weighted residual. (**c**) Preference for novel faces does not predict later plasma OT concentrations (P = 0.2138); as before, the y axis values (plasma OT level) are corrected. (**d**) Plasma OT concentrations do not predict CSF OT concentrations (P = 0.8674), as before the y axis (CSF OT level) is corrected. In panels (**b**,**c**, & **d**), the line depicts the expected values for each data point from the WLS-GLM regression equation.

As part of a colony-wide infant behavioural assessment program, our subjects (N = 60; 3–4 month old infant males) underwent a standard paired-comparison face recognition test (see Methods). In this task, the subject’s unequal distribution of visual attention towards an unfamiliar vs. familiar face (i.e., preference for novel faces) was used to infer the ability to recognize a familiar face^39^. As expected, we found naturally occurring variation in the proportion of time that infants spent gazing at novel face stimuli (Fig. 1a).

### Early preference for novel faces predicts later CSF but not blood OT concentrations

CSF and blood OT concentrations were quantified using standard procedures (see Methods). Since blood OT does not have access to relevant receptor-expressing neural substrates^37^, we predicted that our measure of social perception would be most strongly associated with CSF OT concentrations. We thus first confirmed that the effect of preference for novel faces was due to the duration spent attending to the novel face, rather than differences in time spent attending to faces overall, by including the log_10_ of each measure as separate variables in a Weighted Least Squares-General Linear Model (WLS-GLM) predicting CSF OT concentration. As expected, duration attending to a novel face predicted CSF OT concentration (WLS-GLM: F_1,53_ = 9.9044; partial r = 0.40; β_1_ = 40.65 ± 12.92; P = 0.0027) despite controlling for total time attending to both faces. As would be expected if this second variable is important as a divisor for the first, it also showed a weaker reciprocal relationship (WLS-GLM: F_1,53_ = 4.4742; partial r = −0.28; β_2_ = −29.92 ± 14.15; P = 0.0391). We therefore performed all subsequent analyses representing preference for novel faces as the time spent attending to novel faces divided by the time spent attending to any face. As predicted, preference for novel faces positively predicted CSF OT concentrations (WLS-GLM: F_1,54_ = 8.6404; partial r = 0.37; β_1_ = 26.40 ± 8.980; P = 0.0048; Fig. 1b), but not plasma OT concentrations (WLS-GLM: F_1,37_ = 1.5997; partial r = −0.20; β_1_ = −2.635 ± 2.083; P = 0.2138; Fig. 1c), and did so in subjects ranging in age from juvenility to adulthood.

### Plasma and CSF OT concentrations are unrelated within individuals

We next assessed the utility of using peripheral OT assessment as a surrogate for central OT assessment. We did so by testing whether plasma OT concentrations positively predicted CSF OT concentrations in concomitantly collected blood and CSF samples. Given that preference for novel faces predicted CSF, but not plasma, OT concentrations, we hypothesized that these OT measurements would be unrelated to one another. In keeping with this hypothesis, we did not find a relationship between plasma and CSF OT concentrations within individuals (WLS-GLM: F_1,54_ = 0.0282; partial r = −0.02; β_1_ = −0.09571 ± 0.5704; P = 0.8674; Fig. 1d).

## Discussion

The ability to recognize faces of one’s own species has been experimentally confirmed (via visual paired-comparison or match-to-sample paradigms) across primate taxa and includes reports from humans (*Homo sapiens*)^40,41^, chimpanzees (*Pan troglodytes*)^2^, orangutans (*Pongo* spp.)^42^, capuchin monkeys (*Cebus apella*)^41^, and Japanese, tonkean, and rhesus macaques (*Macaca fuscata, tonkeana*, and *mulatta)* ^2,41,43^. This ability appears within the first few months of life, and develops regardless of prior exposure to faces^9,44^. In visually-dependent primate species, face recognition is critical for the formation of long-lasting social relationships and is thought to have enabled the evolution of large, socially complex primate groups^1,4^. Despite the crucial role of face recognition in primate societies, there nevertheless exists significant variation in performance on tasks used to assess face recognition ability, a phenomenon replicated here (Fig. 1a)^8,9^. This variation has long-lasting consequences for social development, as infant rhesus monkeys that have poor face recognition skills spend more time alone and less time engaged in social interactions later in life^10^.

In both human and non-human primates, it is well documented that intranasal OT administration influences how faces are processed^12^. For example, OT administration promotes attention to the eye region^17,22^ and to faces^18,20^ while selectively decreasing attention to threatening faces^19^. OT administration also improves the ability of people to correctly identify familiar faces^23,24,26^, and to identify individuals by face^25,26^. Evidence from the present study therefore extends these pharmacological findings by providing an important physiological validation of OT’s role in face processing. Specifically, we found that individual variation in preference for novel faces significantly and positively predicted endogenous CSF OT concentrations, and did so up to five years after behavioural assessment (Fig. 1b). These results are also consistent with the idea that individual recognition abilities critical for group living may be supported by central OT biology^45^.

Although we observed a predictive relationship between early performance in a task that measures face recognition ability and later CSF OT concentrations, the available data do not allow us to conclude the direction of the relationship between these two variables. Given that the rodent literature has experimentally established a causal relationship between OT and social recognition^14,45–47^, it is reasonable to hypothesize that early and putatively stable differences in central OT function underlie the observed variation in infant primate performance on a face recognition task. However, it is also possible that other variables, such as early parental interactions (which are thought to be capable of influencing social perception^48,49^ and peripheral levels of OT^50^), may explain both early differences in the ability to recognize faces as well as later central OT function. Similarly plausible is the notion that early variation in infant social perceptual ability leads to different socialization patterns during development, which, in turn, produced variation in the observed CSF OT concentrations later in life. Research involving CSF sampling earlier in life and pharmacological OT receptor blockade is now required to address the developmental stability of CSF OT concentrations as well as to determine the causal role of central OT signalling pathways in primate face recognition ability.

Unlike for our central measure of OT biology, preference for novel faces did not predict blood OT concentrations in these subjects (Fig. 1c). There has been much debate over the functional significance of peripheral OT measurement. Although many studies have reported positive relationships between social behaviour and peripheral OT measurements in human and non-human primates^16,51–53^, including some of our own patient studies^54,55^, synthesis and interpretation of this collective evidence has been complicated by multiple factors. These factors include the following: 1) peripheral OT has been variously measured in blood, saliva, and urine samples; 2) studies have employed different measurement techniques (e.g., enzyme immunoassay or radioimmunoassay), with or without solid phase extraction, thereby yielding strikingly different OT values; 3) peripheral OT concentrations have been assessed under basal as well as stimulated conditions; 4) social functioning has been assessed using both trait and state measurements; 5) study samples have varied from healthy human and non-human primates to case-control clinical comparisons; and 6) studies have been conducted across multiple species^38,56^. Systematic research on peripheral OT measurement (using gold-standard OT quantification techniques), including careful evaluation of specific biological media in the context of both state and trait social functioning assessments, within a well-defined study population, is urgently needed.

Perhaps the largest gap in knowledge with regard to peripheral OT is that few prior studies have assessed both peripheral and central OT measurements during the same sampling session to compare which measurement is more informative within the context of behavioural assessment^57^. A strength of the present study is that we concomitantly collected blood and CSF samples, evaluated them both in the context of a face recognition ability task (as discussed above), and found that blood OT concentrations were unrelated to CSF OT concentrations within individuals (Fig. 1d). Although CSF OT may play a regulatory role in the control of peripheral oxytocin concentrations^36^, and our group has previously reported that blood OT concentrations robustly and positively predict CSF OT concentrations in humans (with both OT measures inversely related to anxiety)^58^, other research suggests that the central and peripheral OT pathways may be functionally independent^59–61^. Indeed, a previous primate study found that maternally-deprived rhesus monkeys showed lower CSF OT concentrations compared to control mother-reared monkeys, but blood OT levels in these two groups were indistinguishable^57^. The present and currently available findings therefore lead us to conclude that CSF OT concentrations are a more valid measure by which to assess social perception traits in rhesus monkeys than blood OT concentrations.

We note that this study was performed on a cohort of exclusively male rhesus macaques. Given documented sex differences in rhesus infant facial perception and response to OT administration (i.e., male infants look less at conspecific faces compared to female infants^62^ and administration of intranasal OT improves infant male, but not female, gaze following abilities^63^), our results establishing a relationship between performance on a face recognition task and later CSF OT concentrations may be sex-specific as well. Similarly, the OT system is well known to be behaviourally and functionally sexually dimorphic^36,45,51,64^. Research on female rhesus monkeys is now needed to systematically understand some of these potential sex differences in social perception and related neurobiology.

We also note that the visual-paired comparison task used in this study has its own constraints and limitations. For example, variation in preference for novel faces could be influenced by a variety of internal factors (e.g., anxiety or motivation) which could contribute to attention and gaze distribution. To guard against this, analyses in which attention to novel faces and overall attention to faces were initially evaluated in our model as separate variables to rule out the possibility that the preference for novel faces was due to these internal mental states. It is remains possible, although not parsimonious, that variation in any such factors could be driving the relationship between preference for novel faces and CSF OT concentration.

Finally, a question that remains unanswered by our study is the domain specificity of our findings. For example, would CSF OT concentrations also have predicted performance on an identical task that used non-social objects instead of pictures of monkey faces (i.e., therefore indicating a global preference for novel stimuli), or would CSF OT concentrations only have predicted aspects of behavioural functioning related to social perception? Unfortunately, this study was not designed to address this question; follow-up research that explicitly tests this hypothesis is required.

In conclusion, these findings are the first to show an association between face processing and endogenous brain (but not blood) OT biology in primates. These data underscore the importance of measuring CSF rather than blood OT concentrations in studies of primate social cognition. The fact that performance on a task that measures face recognition predicted CSF OT concentrations years after behavioural testing suggests that central OT biology may support individual recognition abilities critical for group living. Finally, by revealing that natural variation in preference for novel faces predicts CSF OT concentrations in rhesus macaques, we continue to better understand the neurobiological substrates underlying a critical cognitive step in the evolution of social groups^35^.

## Methods

### Subjects and study site

Subjects were N = 60 male rhesus monkeys (*Macaca mulatta*) that were born and reared at the California National Primate Research Centre (CNPRC). Subjects lived in outdoor, half-acre (0.19 ha) field corrals, measuring 30.5 m wide × 61 m deep × 9 m high. Each corral contained up to 150 animals of mixed age and sex social groups. Monkeys had *ad libitum* access to Lixit-dispensed water, primate laboratory chow was provided twice daily, and fruit and vegetable supplements were provided twice weekly. Various toys, swinging perches, along with outdoor and social housing, provided a stimulating environment. All procedures were approved by institutional IACUCs and complied with NIH policies on the care and use of animals.

### Infant BioBehavioural Assessment (BBA) program

Subjects were enrolled in the colony-wide BBA program at an average of 105 ± 3 days of age (CI: 99%; range: 90–127 days). The BBA program consists of a battery of tests designed to assess infants’ behavioural and physiological reactivity as described in detail in previous publications^65–67^. One of these assessments, the face recognition test, provided a means by which to investigate individual differences in preference for novel faces in the present study.

During BBA testing, infants were removed from their home cages and separated from their mothers for a 25 h period. BBA testing occurred in cohorts of five to eight monkeys at a time, drawn from multiple social groups. During testing, subjects were housed individually in standard-sized holding cages (39 × 52 × 47 cm), and each infant was individually assessed according to a predetermined random order. Tests were video-recorded and coded at a later date. Immediately following the completion of BBA testing, infants were reunited with their mothers, and one hour later, returned to their home corrals.

### Face recognition test

The face recognition test, adapted from prior published studies^68,69^, consisted of still colour photographs of rhesus monkey faces projected onto a monitor (32″ Panasonic KV 32540) in front of the infant subject (See Supplementary Video S1). Stimuli were neutral faces of unfamiliar individuals of different ages (i.e., adult and juvenile) and sex. The software package ‘Cortex’ was used to program the stimuli. Once programmed, the stimulus sequence was played back on a computer monitor and recorded to create a stimulus DVD.

During testing, two pictures (each measuring 19.7 × 22.9 cm) were always presented simultaneously, with each picture occupying either the left or right third of the screen. A low-light camera (Radio Shack Observation 49–2502), attached to the playback monitor and situated midway between the two projected images, was used to record the subjects’ looking responses. Each subject was administered seven problem sets, with each problem comprising one 20-second “familiarization” and two 8-second “recognition” trials. During a familiarization trial, the subject was presented with a pair of identical rhesus monkey faces. Following a brief 5-second delay, the participant was then simultaneously presented, during the first recognition trial, with the now familiar face and a novel face. A second recognition trial was conducted identically, except that the positions of the novel and familiar face were reversed in order to avoid directional bias. A proportional preference for novel faces compared to the familiar faces indicates a subject’s face recognition ability.

As previously described in a published manuscript^10^, four measures of looking behaviour were scored using Observer XT software (Noldus Inc., Leesburg, VA, USA). The four measures coded for each trial were duration of gaze: 1) directed to the left stimulus, 2) directed to the right stimulus, 3) directed elsewhere (but determinable), and 4) not determinable. All videos were coded by a single observer. Intra-observer (test-retest) reliability was assessed annually by coding up to 15 videos per year on two occasions at least several weeks apart. Annual reliability values, calculated as the percent agreement (line by line) of the two codings, range from 86.1–92.2%. The duration of time gazing on target was calculated as the sum of 1 and 2. For the recognition trials, the durations for 1 and 2, above, were recoded as duration attending to familiar and novel faces. These data were summed across both recognition trials, to give the total duration attending to a novel face, and the total duration on target for each problem.

Each subject, thus, yielded data for seven problem sets. As the problem sets might differ systematically in salience of individual stimuli, we followed our previous approach^10^, and ran a Repeated Measures-General Linear Model (RM-GLM) to obtain a least squares mean for each subject, for both duration attending to a novel face, and duration on target (i.e., a session corrected mean). This approach yields mean scores corrected for both systematic differences between problem sets, and for recognition trials in which a subject fails to attend to either face.

### Sample collection and processing

CSF and blood samples were concomitantly obtained from each monkey subject to allow for direct comparison of the two matrices. Subjects underwent sample collection during one of two collection sessions when they were between 1 and 5 years of age. Samples were collected between 9–11 AM to minimize any potential circadian effects on OT concentrations. Each subject was captured from his home corral, rapidly immobilized with telazol (5–8 mg/kg), and moved to an indoor procedure room. Supplementary ketamine (5–8 mg/kg) was used as needed to maintain complete immobilization. Collection of both CSF and blood samples was accomplished within 10–15 min of initial cage entry. CSF (2 mL) was drawn from the cisterna magna using standard sterile procedure. Cisternal CSF sampling is attractive because it prevents the loss in signal of brain-released neurotransmitters typically associated with the more distal lumbar sampling procedures utilized in humans^70^. CSF samples were immediately aliquoted into 1.5 mL siliconized polypropylene tubes and flash-frozen on dry ice. Immediately following CSF collection, whole blood samples (up to 25 mL) were drawn from the femoral vein, dispensed into EDTA-treated vacutainer tubes, and placed on wet ice. Whole blood samples for neuropeptide quantification were promptly centrifuged (1600 × *g* at 4 °C for 15 min), and the plasma fraction was aliquoted into 1.5 mL polypropylene tubes and flash-frozen on dry ice. All samples were stored at −80 °C until quantification. After sample collection, each subject was administered replacement fluids and ketoprofen as needed. Each subject was placed in a standard laboratory cage for recovery overnight, after which point he was returned to his home corral.

### OT quantification

CSF and blood OT concentrations were quantified using a commercially available enzyme immunoassay kit (Enzo Life Sciences, Farmingdale, NY). This kit has been validated for use in rhesus monkeys and is highly specific and exclusively recognizes OT and not related peptides (i.e., the OT cross-reactivity with arginine vasopressin is 0.6%). The assay’s detection limit is 11.7 pg/mL. A trained technician blinded to experimental conditions performed sample preparation and OT quantification. The CSF samples were directly assayed for OT using established protocols^71,72^, and the plasma samples were extracted using methods recommended by the manufacturer, and as previously published^54,55^. All CSF and plasma samples were assayed in duplicate (100 µL per well) with a tuneable microplate reader for 96-well format according to manufacturer’s instructions.

### Statistical analyses

We have previously observed individual variation in preference for novel faces^10^. The following analyses tested if this individual variation was meaningful in terms of predicting OT concentrations later in life. All analyses were performed in JMP Pro 13.0 and SAS 9.4 for Windows (SAS Institute Inc., Cary, NC). Diagnostics of early analyses suggested that larger predicted OT values were associated with larger residuals and leverage. Statistically, this violates the underlying assumptions of general linear methods, and runs the risk that extreme values may drive the results. OT assay values are derived from means of individual wells, and all things being equal, one would expect the variance to increase with the mean. Accordingly, larger OT assay means in general had higher coefficient of variation (CV) values. Rather than reject means above some critical CV (which would involve systematically biasing against high mean values), we adopted a more powerful approach, in which the relationship between each mean and CV is recognized and included in the analysis. To do so, we used WLS-GLM, in which the weighting of each data point systematically controls the impact of less reliable but more extreme data points^73^. The ideal term to use for weighting in these models is the reciprocal of the estimated variance of the data point, which is notoriously difficult to obtain^73^, and thus, this powerful theoretical approach is rarely used in practice. However, in this case, as each assay value is a mean with an associated CV, the variance can be estimated directly. The CV for each data point was thus converted to a variance, and then into a weight as the reciprocal of this variance.

Subject date of birth was initially included in our model as a blocking factor. After verifying its lack of influence on the model, and given the collinearity with collection session, subject date of birth was excluded in order to avoid model overspecification. Every analysis was then performed as a WLS-GLM, with the collection session included as a blocking factor (the same results held if collection session was omitted, but inclusion of this variable in the model is the more conservative approach). To test whether preference for novel faces predicted CSF OT concentrations later in life, we performed a WLS-GLM predicting mean CSF OT concentrations, weighted by the reciprocal of the between-well variance, and blocking by collection session. Typically face recognition tasks calculate the preference for novel faces as the duration of time attending to a novel face divided by the total time spent on target. However, doing so potentially masks the fact that these two measures convey different information about the task. The total duration of time attending to both faces captures many alternative factors impacting task performance, such as anxiety or motivation. The duration spent attending to the novel face is likely affected by these measures, and also by preference for novelty and for novel faces. We therefore first performed an analysis in which both measures were included as separate variables. These variables were logged, exploiting the fact that log(a) − log(b) = log(a/b), so that the regression equation would model the simpler combined variable (in which duration attending to novel faces is divided by total duration attending to both faces). This initial analysis confirmed that duration attending to novel faces was positively correlated with CSF OT concentration, and that total duration attending to both faces in the same model was reciprocally correlated. This result rules out the possibility that any preference is due to an alternative variable (like motivation), and justifies the use of the simpler combined variable. However, the resulting multiple regression cannot be plotted in an easily understandable way. Thus, we ultimately calculated the duration attending to novel faces divided by the duration attending to both faces, and used this simpler single variable in all analyses. In these analyses, one animal did not yield usable behavioural data, one animal did not yield usable CSF samples, and one animal only yielded one good sample well (and thus a CV could not be calculated). These three animals were excluded.

To test whether preference for novel faces predicted plasma OT concentrations later in life, we performed the same analysis, but using the mean and variance of plasma OT wells. Several plasma samples yielded only one usable well, and thus no CV or variance could be calculated. These individuals were excluded from this analysis.

To test if plasma OT concentrations predicted CSF OT concentrations we performed the same analysis, using the mean and variance of the CSF OT wells. We included all individuals with plasma OT data, regardless of CV, with the exception of one individual which had a biologically implausible plasma value. Including or excluding this individual had no effect on the results.

The assumptions of WLS-GLM (linearity, homogeneity of variance, and normality of error) were confirmed graphically post-hoc^74^. Data are plotted as the expected value for each data point (controlling for collection session), plus the weighted residual. Thus the Y axis is corrected for both collection session, and the CV of the original sample. Effect sizes are reported as partial r (i.e., correlation coefficients, which are unitless and scale-invariant), and also as the regression coefficient (the Beta, β_1_) ± its standard error (which are absolute effects sizes in arbitrary units equivalent to the units of y divided by the units of x).

### Data availability

The corresponding data for this manuscript are available as supplemental information.

## Electronic supplementary material

Supplementary Video S1

Supplementary Dataset
