# Supplementary material for: Preference for novel faces in male infant monkeys predicts cerebrospinal fluid oxytocin concentrations later in life
Source: Sci Rep. 2017 Oct 11;7:12935. doi: 10.1038/s41598-017-13109-5 (PMC5636831; doi:10.1038/s41598-017-13109-5)
Supplement: Supplementary file 2 — Supplementary Dataset [file 41598_2017_13109_MOESM2_ESM.doc]

Preference for novel faces in male infant monkeys predicts cerebrospinal fluid oxytocin concentrations later in life

Jesus E. Madrid^*^, Ozge Oztan, Valentina Sclafani, Laura A. Del Rosso, Laura A. Calonder, Katie Chun, John P. Capitanio, Joseph P. Garner, and Karen J. Parker^*^

**Supplementary Video S1.** **Face recognition test sample stimuli**. Preference for novel faces is assessed via a visual paired-comparison test. Stimuli consists of neutral faces of unfamiliar rhesus macaques of mixed age and sex. Each subject is administered seven problem sets. Each problem set is preceded by the presentation of a blank screen, followed by a familiarization trial, and two recognition trials. During the familiarization trial the identical faces are simultaneously presented in two halves of the screen. During the proceeding first recognition trial, the now familiar face is presented on only one half of the screen, and a novel face presented on the other half (with the location determined randomly). During the second recognition trial, the stimulus positions are reversed in order to avoid directional bias. A tone emanating from the monitor’s speaker is presented to facilitate the subjects’ orientation towards the monitor and a low-light camera, attached to the monitor and situated midway between the two face stimuli, is used to record the subjects’ looking responses.

# Supplementary Dataset.

Analysis 1: GLM multiple regression of log-transformed durations that make up the preference for novelty measure (Novel stimulus, and Total time on target), predicting CSF OT concentration

**DATA** Analysis_1;

INPUT Collection &$16. OXT_CSF inv_OXT_var log10_Total_Novel_sessioncontrol log10_TotalLooking_sessioncontro; Lines;

COLLECTION01 46.761 0.005437965 -1.47664379334521 -

0.795418824422429

| COLLECTION01 46.013 0.031219697 -0.741518363362603 - | | | | | |
| --- | --- | --- | --- | --- | --- |
| 0.468775219571868 | |  | | | |
| COLLECTION01 | 61.547 | | 0.002643699 | -0.556413173101531 | - |
| 0.282616292589276 | |  | | | |
| COLLECTION01 | 50.857 | | 0.001452109 | -0.274034492408039 | - |

0.109001814711141

COLLECTION01 86.68 0.000587419 -0.913700689986153 -

| 0.728942921894913 | |  | | | |
| --- | --- | --- | --- | --- | --- |
| COLLECTION01 | 41.029 | | 0.015783913 | -0.926585945618056 | - |
| 0.725652996625845 | |  | | | |

COLLECTION01 46.967 0.005070748 -0.58166721518418 -

0.363788113764844

COLLECTION01 43.645 0.026405402 -0.528517593372337 -

0.375864206613168

COLLECTION01 53.034 1.097352661 -0.566182180385687 -

0.45007429047052

COLLECTION01 39.24 0.010559385 -0.706582195052248 -

| 0.160038150408262 | |  | | | |
| --- | --- | --- | --- | --- | --- |
| COLLECTION01 | 27.207 | | 0.059249514 | -0.665423618185473 | - |
| 0.423633883043223 | |  | | | |

COLLECTION01 84.82 0.000365118 -0.697565164827689 -

0.406206481700867

| COLLECTION01 46.939 0.009126886 -0.627156090909555 - | | | | | |
| --- | --- | --- | --- | --- | --- |
| 0.478423395688432 | |  | | | |
| COLLECTION01 | 41.647 | | 0.004473461 | -0.286012444825263 | - |

0.076213089954664

COLLECTION01 61.653 0.003983138 -1.36738076338088 -

0.697462365166119

| COLLECTION01 42.078 0.003295256 -0.841016669487038 - | | | | | |
| --- | --- | --- | --- | --- | --- |
| 0.552878264784735 | |  | | | |
| COLLECTION01 | 37.623 | | 0.001545965 | -0.657758971218301 | - |

0.288554862040706

COLLECTION01 59.596 0.018311436 -0.52148112069714 -

0.414149482861161

COLLECTION01 35.517 0.112348914 -0.681046022457549 -

0.521472462801365

COLLECTION01 40.439 0.09320284 -0.660994013445996 -

0.515268216919898

COLLECTION01 51.123 0.003233333 -0.401293734845231 -

0.19957685711467

COLLECTION01 58.479 0.000840033 -0.343011114009931 -

0.208818875822672

COLLECTION01 39.297 0.022145678 -0.797007932054064 -

0.615509848542171

COLLECTION01 48.617 0.017839482 -0.576798377189274 -

0.350509660538166

COLLECTION01 45.748 53.09006605 -0.751644134198632 -

0.531332975191042

COLLECTION01 50.661 0.001778938 -0.863117791259853 -

0.462831322863849

COLLECTION01 51.861 0.005948921 -0.604757502901271 -

0.371336069800478

COLLECTION01 27.563 0.016909806 -0.850688494092085 -

0.530136470397915

COLLECTION01 53.679 0.037657259 -0.222215159153842 -

0.0239666164312447

COLLECTION02 31.343 0.012710013 -0.620924837272545 -

0.361754715334867

COLLECTION02 19.798 0.183228635 -0.759627999998763 -

0.461507075606598

COLLECTION02 44.112 0.375390307 -0.981740675675525 -

0.730600080450039

COLLECTION02 57.976 0.00086343 -0.471495946968091 -

0.136252393475472

COLLECTION02 33.616 0.066913269 -0.402644925219685 -

0.199384223098682

COLLECTION02 43.103 0.165666727 -0.893979180859731 -

0.683144973131537

COLLECTION02 40.793 0.142233532 -0.587420184970777 -

0.31634195560007

COLLECTION02 28.419 0.073264858 -0.680806465719829 -

0.511188080351988

COLLECTION02 32.434 0.013536526 -0.48353866739553 -

0.278765557244495

COLLECTION02 25.429 0.095881318 -0.775060543018984 -

0.546517912872305

COLLECTION02 17.118 0.047870882 -0.612736564625664 -

0.38608273789797

COLLECTION02 31.182 0.002110966 -0.644104259322084 -

0.269936114924355

COLLECTION02 22.944 0.676254227 -0.814194458709971 -

0.399883148377969

COLLECTION02 33.204 0.013840101 -0.964754425707032 -

0.530258372745199

COLLECTION02 26.518 0.408521054 -1.12452201390058 -

0.879548478124812

COLLECTION02 42.769 0.004566565 -0.281770764817507 -

0.0656439297845148

COLLECTION02 31.535 0.011713295 -0.426280377970925 -

0.232318547446364

COLLECTION02 34.292 0.001578419 -0.484078492436446 -

0.245614659082272

COLLECTION02 27.967 0.014892685 -0.521846352474103 -

0.29917267359203

COLLECTION02 13.193 0.084339663 -0.438345362746496 -

0.0771078090865275

COLLECTION02 36.677 0.003147312 -0.363834182696907 -

0.105624613639157

COLLECTION02 49.24 0.25777686 -0.385705099865176 -

0.0499134364121381

COLLECTION02 33.058 0.01452444 -0.492432854752245 -

0.203128422504012

COLLECTION02 19.38 0.224098763 -0.807672338944973 -

0.63726181165191

COLLECTION02 33.66 0.045031427 -0.397279298161685 -

0.126064147638295

COLLECTION02 39.327 1.791064235 -0.20176866143968

0.00516519176615116

COLLECTION02 31.625 0.004164346 -0.516973583057497 -

0.361510743045363

COLLECTION02 43.079 0.005535535 -0.50366410900529 -

0.255427671494051

;

**RUN**;

**PROC GLM** DATA=Analysis_1 ; WEIGHT inv_OXT_var;

CLASS Collection;

MODEL OXT_CSF = Collection log10_Total_Novel_sessioncontrol log10_TotalLooking_sessioncontro/ solution SS3 ;

**RUN**;

# Analysis 2 (Figure 1b): Analysis of the same data in analysis 1, but now, preference for novelty is figured as a straight ratio of time attending to the novel stimulus over total time on target

**DATA** Analysis_2_Fig1b;

INPUT Collection &$16. OXT_CSF NovelPreferredOverTotalLooking

| inv_OXT_var;  Lines; |  | | |
| --- | --- | --- | --- |
| COLLECTION01 | 46.761 | 0.08068616 | 0.005437965 |
| COLLECTION01 | 46.013 | 0.43666704 | 0.031219697 |
| COLLECTION01 | 61.547 | 0.54763357 | 0.002643699 |
| COLLECTION01 | 50.857 | 0.67398923 | 0.001452109 |
| COLLECTION01 | 86.68 | 0.44789776 | 0.000587419 |
| COLLECTION01 | 41.029 | 0.67783437 | 0.015783913 |
| COLLECTION01 | 46.967 | 0.44605156 | 0.005070748 |
| COLLECTION01 | 43.645 | 0.60423678 | 0.026405402 |
| COLLECTION01 | 53.034 | 0.79533878 | 1.097352661 |
| COLLECTION01 | 39.24 | 0.2739136 | 0.010559385 |
| COLLECTION01 | 27.207 | 0.56985013 | 0.059249514 |
| COLLECTION01 | 84.82 | 0.5395066 | 0.000365118 |
| COLLECTION01 | 46.939 | 0.75169347 | 0.009126886 |
| COLLECTION01 | 41.647 | 0.61051134 | 0.004473461 |
| COLLECTION01 | 61.653 | 0.36297942 | 0.003983138 |
| COLLECTION01 | 42.078 | 0.47908802 | 0.003295256 |
| COLLECTION01 | 37.623 | 0.4977626 | 0.001545965 |
| COLLECTION01 | 59.596 | 0.75096366 | 0.018311436 |
| COLLECTION01 | 35.517 | 0.69176473 | 0.112348914 |
| COLLECTION01 | 40.439 | 0.69765133 | 0.09320284 |
| COLLECTION01 | 51.123 | 0.64356943 | 0.003233333 |
| COLLECTION01 | 58.479 | 0.66619917 | 0.000840033 |
| COLLECTION01 | 39.297 | 0.49854736 | 0.022145678 |
| COLLECTION01 | 48.617 | 0.55357468 | 0.017839482 |
| COLLECTION01 | 45.748 | 0.58616729 | 53.09006605 |
| COLLECTION01 | 50.661 | 0.52350335 | 0.001778938 |
| COLLECTION01 | 51.861 | 0.76608285 | 0.005948921 |
| COLLECTION01 | 27.563 | 0.53648328 | 0.016909806 |
| COLLECTION01 | 53.679 | 0.61731402 | 0.037657259 |
| COLLECTION02 | 31.343 | 0.46283235 | 0.012710013 |
| COLLECTION02 | 19.798 | 0.30581361 | 0.183228635 |
| COLLECTION02 | 44.112 | 0.32718157 | 0.375390307 |
| COLLECTION02 | 57.976 | 0.46134802 | 0.00086343 |
| COLLECTION02 | 33.616 | 0.64443417 | 0.066913269 |
| COLLECTION02 | 43.103 | 0.5452518 | 0.165666727 |
| COLLECTION02 | 40.793 | 0.47774569 | 0.142233532 |
| COLLECTION02 | 28.419 | 0.63505619 | 0.073264858 |
| COLLECTION02 | 32.434 | 0.60580957 | 0.013536526 |
| COLLECTION02 | 25.429 | 0.45236556 | 0.095881318 |
| COLLECTION02 | 17.118 | 0.50393443 | 0.047870882 |
| COLLECTION02 | 31.182 | 0.48501294 | 0.002110966 |

| COLLECTION02 | 22.944 | 0.36145862 | 0.676254227 |
| --- | --- | --- | --- |
| COLLECTION02 | 33.204 | 0.33483056 | 0.013840101 |
| COLLECTION02 | 26.518 | 0.44970542 | 0.408521054 |
| COLLECTION02 | 42.769 | 0.52386566 | 0.004566565 |
| COLLECTION02 | 31.535 | 0.44042287 | 0.011713295 |
| COLLECTION02 | 34.292 | 0.45860462 | 0.001578419 |
| COLLECTION02 | 27.967 | 0.48655286 | 0.014892685 |
| COLLECTION02 | 13.193 | 0.40582762 | 0.084339663 |
| COLLECTION02 | 36.677 | 0.52770296 | 0.003147312 |
| COLLECTION02 | 49.24 | 0.45672143 | 0.25777686 |
| COLLECTION02 | 33.058 | 0.49218647 | 0.01452444 |
| COLLECTION02 | 19.38 | 0.6562946 0.224098763 | |
| COLLECTION02 | 33.66 | 0.52388912 0.045031427 | |
| COLLECTION02 | 39.327 | 0.56885229 | 1.791064235 |
| COLLECTION02 | 31.625 | 0.63662224 | 0.004164346 |
| COLLECTION02 | 43.079 | 0.69061493 | 0.005535535 |
| ;  **RUN**; |  |  |  |

**PROC GLM** DATA=Analysis_2_Fig1b; WEIGHT inv_OXT_var;

CLASS Collection;

MODEL OXT_CSF = Collection NovelPreferredOverTotalLooking/ ss3 solution;

**RUN**;

# Analysis 3 (Figure 1c): Does preference for novelty as figured as a straight ratio of time attending to the novel stimulus over total time on target, predict plasma OXT concentration?

**DATA** Analysis_3_Fig1c;

INPUT Collection &$16. OXT_Plasma NovelPreferredOverTotalLooking inv_OXT_Blood_var;

Lines;

| COLLECTION01 | 12.801 | 0.08068616 | 1.489883316 |
| --- | --- | --- | --- |
| COLLECTION01 | 2.273 | 0.54763357 | 0.242715891 |
| COLLECTION01 | 13.09 | 0.67398923 | 0.049605801 |
| COLLECTION01 | 8.233 | 0.44789776 | 7.620411368 |
| COLLECTION01 | 7.554 | 0.44605156 | 0.083180336 |
| COLLECTION01 | 10.08 | 0.60423678 | 0.060002437 |
| COLLECTION01 | 8.981 | 0.79533878 | 0.258501089 |
| COLLECTION01 | 6.782 | 0.2739136 | 0.142940473 |
| COLLECTION01 | 14.905 | 0.5395066 | 1.664674596 |
| COLLECTION01 | 7.604 | 0.61051134 | 0.265971696 |
| COLLECTION01 | 2.942 | 0.36297942 | 0.916764858 |
| COLLECTION01 | 6.239 | 0.47908802 | 0.101136741 |
| COLLECTION01 | 10.944 | 0.4977626 | 0.202607611 |
| COLLECTION01 | 11.663 | 0.69176473 | 0.765468454 |
| COLLECTION01 | 6.537 | 0.64356943 | 3.316534031 |
| COLLECTION01 | 6.212 | 0.66619917 | 0.096206051 |
| COLLECTION01 | 11.59 | 0.49854736 | 0.155219006 |
| COLLECTION01 | 7.807 | 0.55357468 | 0.825264979 |
| COLLECTION01 | 7.965 | 0.58616729 | 157.6262123 |
| COLLECTION01 | 7.369 | 0.52350335 | 4603.868343 |
| COLLECTION01 | 8.131 | 0.76608285 | 2.838352679 |
| COLLECTION01 | 6.198 | 0.53648328 | 0.459560763 |
| COLLECTION01 | 12.322 | 0.61731402 | 0.173208168 |
| COLLECTION02 | 3.212 | 0.46283235 | 22.25159033 |
| COLLECTION02 | 5.743 | 0.30581361 | 83.98759891 |
| COLLECTION02 | 5.049 | 0.32718157 | 17.75797919 |
| COLLECTION02 | 11.181 | 0.64443417 | 0.316405815 |
| COLLECTION02 | 6.719 | 0.5452518 | 3.065863862 |
| COLLECTION02 | 4.278 | 0.47774569 | 0.087996623 |
| COLLECTION02 | 7.71 | 0.50393443 | 0.25870861 |
| COLLECTION02 | 6.714 | 0.48501294 | 6.827907278 |
| COLLECTION02 | 5.109 | 0.33483056 | 2.232469855 |
| COLLECTION02 | 2.322 | 0.52386566 | 0.307208715 |
| COLLECTION02 | 5.205 | 0.45860462 | 0.078894428 |
| COLLECTION02 | 4.64 | 0.48655286 | 4.644768133 |
| COLLECTION02 | 5.061 | 0.52770296 | 0.634781065 |
| COLLECTION02 | 7.708 | 0.45672143 | 0.052354073 |
| COLLECTION02 | 7.413 | 0.52388912 | 2.70635208 |
| COLLECTION02 | 5.789 | 0.63662224 | 0.349967083 |
| COLLECTION02 | 2.041 | 0.69061493 | 7.247216152 |
| ; |  |  |  |

**RUN**;

**PROC GLM** DATA=Analysis_3_Fig1c; WEIGHT inv_OXT_Blood_var;

CLASS Collection;

MODEL OXT_Plasma = Collection NovelPreferredOverTotalLooking/ ss3 solution;

**RUN**;

# Analysis 4 (Figure 1d): Does plasma OXT concentration predict CSF OXT concentration?

**DATA** Analysis_4_Fig1d;

INPUT Collection &$16. OXT_CSF OXT_Plasma inv_OXT_var; Lines;

| COLLECTION01 46.761 12.801 0.005437965 | | | |
| --- | --- | --- | --- |
| COLLECTION01 | 46.013 | 4.884 | 0.031219697 |
| COLLECTION01 | 61.547 | 2.273 | 0.002643699 |
| COLLECTION01 | 50.857 | 13.09 | 0.001452109 |
| COLLECTION01 | 86.68 | 8.233 | 0.000587419 |
| COLLECTION01 | 41.029 | 7.853 | 0.015783913 |
| COLLECTION01 | 46.967 | 7.554 | 0.005070748 |
| COLLECTION01 | 43.645 | 10.08 | 0.026405402 |
| COLLECTION01 | 53.034 | 8.981 | 1.097352661 |
| COLLECTION01 | 39.24 | 6.782 | 0.010559385 |
| COLLECTION01 | 27.207 | 3.978 | 0.059249514 |
| COLLECTION01 | 84.82 | 14.905 | 0.000365118 |
| COLLECTION01 | 46.939 | 1.082 | 0.009126886 |
| COLLECTION01 | 41.647 | 7.604 | 0.004473461 |
| COLLECTION01 | 61.653 | 2.942 | 0.003983138 |
| COLLECTION01 | 42.078 | 6.239 | 0.003295256 |
| COLLECTION01 | 37.623 | 10.944 | 0.001545965 |
| COLLECTION01 | 90.746 | 14.406 | 0.000454322 |
| COLLECTION01 | 59.596 | 2.101 | 0.018311436 |
| COLLECTION01 | 35.517 | 11.663 | 0.112348914 |
| COLLECTION01 | 40.439 | 3.542 | 0.09320284 |
| COLLECTION01 | 51.123 | 6.537 | 0.003233333 |
| COLLECTION01 | 58.479 | 6.212 | 0.000840033 |
| COLLECTION01 | 39.297 | 11.59 | 0.022145678 |
| COLLECTION01 | 48.617 | 7.807 | 0.017839482 |
| COLLECTION01 | 45.748 | 7.965 | 53.09006605 |
| COLLECTION01 | 50.661 | 7.369 | 0.001778938 |
| COLLECTION01 | 51.861 | 8.131 | 0.005948921 |
| COLLECTION01 | 27.563 | 6.198 | 0.016909806 |
| COLLECTION01 | 53.679 | 12.322 | 0.037657259 |
| COLLECTION02 | 31.343 | 3.212 | 0.012710013 |
| COLLECTION02 | 19.798 | 5.743 | 0.183228635 |
| COLLECTION02 | 44.112 | 5.049 | 0.375390307 |
| COLLECTION02 | 33.616 | 11.181 | 0.066913269 |
| COLLECTION02 | 43.103 | 6.719 | 0.165666727 |
| COLLECTION02 | 40.793 | 4.278 | 0.142233532 |
| COLLECTION02 | 28.419 | 10.902 | 0.073264858 |
| COLLECTION02 | 32.434 | 1.694 | 0.013536526 |
| COLLECTION02 | 29.87 | 5.886 | 0.011153496 |
| COLLECTION02 | 25.429 | 7.154 | 0.095881318 |
| COLLECTION02 | 17.118 | 7.71 | 0.047870882 |
| COLLECTION02 | 31.182 | 6.714 | 0.002110966 |
| COLLECTION02 | 22.944 | 9.856 | 0.676254227 |
| COLLECTION02 | 33.204 | 5.109 | 0.013840101 |

| COLLECTION02 | 26.518 | 2.651 | 0.408521054 |
| --- | --- | --- | --- |
| COLLECTION02 | 42.769 | 2.322 | 0.004566565 |
| COLLECTION02 | 31.535 | 1.657 | 0.011713295 |
| COLLECTION02 | 34.292 | 5.205 | 0.001578419 |
| COLLECTION02 | 27.967 | 4.64 | 0.014892685 |
| COLLECTION02 | 13.193 | 5.777 | 0.084339663 |
| COLLECTION02 | 36.677 | 5.061 | 0.003147312 |
| COLLECTION02 | 49.24 | 7.708 | 0.25777686 |
| COLLECTION02 | 19.38 | 9.273 | 0.224098763 |
| COLLECTION02 | 33.66 | 7.413 | 0.045031427 |
| COLLECTION02 | 39.327 | 7.75 | 1.791064235 |
| COLLECTION02 | 31.625 | 5.789 | 0.004164346 |
| COLLECTION02 | 43.079 | 2.041 | 0.005535535 |
| ;  **RUN**; |  |  |  |

**PROC GLM** DATA=Analysis_4_Fig1d; WEIGHT inv_OXT_var;

CLASS Collection;

MODEL OXT_CSF = Collection OXT_Plasma/ ss3 solution;

**RUN**;
